# Changes in drug-use behaviors among people who use drugs with comorbid psychiatric disorders across the COVID-19 pandemic: a three-phase longitudinal cohort study

**DOI:** 10.3389/fpubh.2026.1878232

**Published:** 2026-09-17

**Authors:** Albert Kar Kin Chung, Johnson Kai Chun Law, Sau Wan Tang, Cheuk Yin Tse, Jerome Jun Hao Chen

**Affiliations:** Department of Psychiatry, Li Ka Shing Faculty of Medicine, The University of Hong Kong, Pokfulam, Hong Kong SAR, China

**Keywords:** comorbid psychiatric disorders, COVID-19, drug-use behaviors, substance misuse, substance use disorder

## Abstract

**Introduction:**

The COVID-19 pandemic substantially affected people who use drugs (PWUD) with comorbid psychiatric disorders. Lockdowns, shifts in drug markets, and fear of infection may have altered drug-use patterns. This prospective study examined changes in drug-use behaviors among PWUD with comorbid psychiatric disorders and identified factors associated with these changes across three distinct phases of the COVID-19 pandemic.

**Methods:**

A cohort of adult PWUD with ICD-10-defined comorbid psychiatric disorders was followed longitudinally from March 2021 to January 2024. Drug-use behaviors were assessed at three time points: during the active COVID-19 pandemic period, after receipt of at least two doses of a World Health Organization (WHO)-endorsed COVID-19 vaccine, and after the WHO declared the end of the global pandemic. Structured interviews and standardized rating scales were used to assess demographic characteristics, ICD-10 comorbid psychiatric disorders, drug-use behaviors, DSM-5 substance use disorder severity, and psychological dependence. COVID-19 infection history and vaccination status were verified through centralized electronic health records. Within-person changes in drug-use behaviors across time points and factors associated with these changes were examined using mixed-effects models.

**Results:**

One hundred and thirty participants (mean age 40.03 years) completed the study. The majority (62.3%) had a comorbid substance-induced psychotic disorder. Overall, 27.7% had laboratory-confirmed COVID-19 infection during the study period. Across the three phases, participants demonstrated significant reductions in substance use disorder severity and drug-use frequency. Sharing of drug-use equipment also declined and remained lower after the pandemic. Greater worry about contracting COVID-19 was associated with increased avoidance of drug use with others and reduced sharing of drug-use equipment. Prior COVID-19 infection was associated with lower drug-use frequency. Among individual drugs, cough syrup was uniquely linked to more frequent use, less solitary use, and sustained reductions in equipment sharing.

**Conclusion:**

Adult PWUD with comorbid psychiatric disorders demonstrated sustained improvements in drug-use behaviors throughout the COVID-19 pandemic and after its official conclusion. Both prior COVID-19 infection and greater worry about contracting COVID-19 were associated with these positive behavioral changes.

## Introduction

Substance use disorder (SUD) represents a major global public health challenge and is associated with substantial health, functional, and social burdens. Access to effective treatment remains limited in many settings (1). Comorbid psychiatric disorders and SUD affect up to 18% of adults and complicate clinical management because of overlapping symptomatology and complex care needs (2). Consequently, people who use drugs (PWUD) with comorbid psychiatric disorders face heightened vulnerability during public health crises. Understanding pandemic-related changes in drug-use behaviors is therefore critical for informing integrated psychiatric and drug rehabilitation services.

The World Health Organization (WHO) declared coronavirus disease 2019 (COVID-19) a global pandemic on 11 March 2020 (3). By the end of March 2025, approximately 778 million confirmed COVID-19 cases and more than 7 million related deaths had been reported globally (4). Beyond the direct health threats and fear of infection, pandemic containment measures involving social distancing and lockdowns disrupted daily routines and adversely affected social interactions, economic stability, and psychological well-being (5, 6).

The unprecedented COVID-19 pandemic had diverse impacts on drug-use behaviors worldwide. Containment measures shifted use from social to solitary contexts among people using opioids, stimulants, cannabis, and hallucinogens (7–10). Changes in the frequency of drug use among PWUD also varied by drug type (8, 11–15). Nevertheless, only a minority of PWUD reported reduced sharing of drugs or drug-use equipment during lockdowns, despite limited drug supplies and heightened risk of COVID-19 transmission (16–18).

Individuals with psychiatric disorders, including SUD, are particularly susceptible to adverse COVID-19 outcomes. This vulnerability stems from compromised cognitive and immune function, unhealthy behaviors, and limited awareness of self-care, even though their risk of infection is comparable to that of the general population (19, 20). Recent meta-analyses also indicate elevated risks of long COVID, COVID-19-associated hospitalization and mortality in this population (21). Although the WHO declared that COVID-19 no longer constituted a global public health emergency in May 2023 (22), up-to-date longitudinal data on changes in drug-use behaviors across the full pandemic trajectory among PWUD with comorbid psychiatric disorders remain scarce.

Given the profound health effects of the COVID-19 pandemic on PWUD and individuals with psychiatric disorders, this prospective cohort study investigated changes in drug-use behaviors and associated factors among PWUD with comorbid psychiatric disorders. The study focused on drugs other than alcohol and examined three critical phases: during the active COVID-19 pandemic period, the period after receipt of ≥2 doses of a WHO-endorsed COVID-19 vaccine, and the period following the WHO’s declaration of the end of the pandemic.

## Materials and methods

### Design

This three-phase, prospective, longitudinal cohort study was conducted at two community-based substance-use treatment clinics operating under the public psychiatric services in Hong Kong from March 2021 to January 2024. Structured interviews and standardized instruments were administered to consenting PWUD with comorbid psychiatric disorders at three pre-specified study time points: during the active COVID-19 pandemic period (T1; baseline commencing March 2021); after receipt of ≥2 doses of a WHO-endorsed COVID-19 vaccine (T2; commencing September 2021); and after the WHO declared the end of the global COVID-19 pandemic (T3; commencing May 2023). This study was approved by the Institutional Review Board of the University of Hong Kong/Hospital Authority Hong Kong West Cluster (HKU/HA HKW IRB UW 21–232).

### Participants

Participants were recruited from two collaborating community-based substance-use treatment clinics through convenience sampling. PWUD aged 18 to 65 years were eligible if they: (a) had used drugs in the three months before the COVID-19 outbreak; (b) continued to use drugs during the pandemic after January 2020; and (c) had a comorbid psychiatric disorder. Because continuous drug use was not required, PWUD with intermittent drug use during the pandemic remained eligible to participate in this study. Individuals were excluded if they: (a) used only alcohol, benzodiazepines, or hypnotics; (b) had used drugs with others on fewer than five occasions in the six months preceding the COVID-19 outbreak; or (c) had no comorbid psychiatric disorder. All participants provided written informed consent before engaging in the study procedures. Each participant received a travel allowance of US$5 after completing all the assessments at each study time point.

### Measures

Baseline demographic characteristics from participants were collected during structured interviews. At each of the three study time points, details on the specific drugs used, frequency of use, number of drugs used, identification of the primary drug, and route of administration were recorded. The substances assessed in the present study comprised seven drugs commonly used in Hong Kong (23, 24): cannabis, cocaine, methamphetamine, cough syrup, heroin, ketamine, and 3,4-methylenedioxymethamphetamine (MDMA; ecstasy). Participants could additionally report other non-listed drugs under the “other” category. The primary drug was defined as the drug most frequently used in the preceding three months. Comorbid psychiatric disorders were diagnosed by board-certified psychiatrists at the study clinics according to the International Classification of Diseases, 10th Revision (ICD-10).

Changes in primary drug-use behaviors were evaluated using self-administered questionnaires consisting of four items: (1) frequency of drug use (1 = abstinent for more than three months, 2 = infrequent, 3 = once a month, 4 = once a week, 5 = twice a week, and 6 = daily); (2) avoidance of using drugs with others (1 = yes, 0 = no); (3) sharing of drug-use equipment with other users (1 = yes, 0 = no); and (4) solitariness of drug use (1 = with others, 2 = mainly with others, 3 = 50:50, 4 = mainly alone, and 5 = alone). Participants were also asked to rate their level of worry about contracting COVID-19 (0 = not at all, 1 = slightly worried, 2 = worried, and 3 = quite worried).

The severity of SUD was determined by the attending psychiatrists during clinic visits according to the Diagnostic and Statistical Manual of Mental Disorders, Fifth Edition (DSM-5), using the Structured Clinical Interview for DSM-5 Disorders—Clinician Version (SCID-5-CV) (25). Drug dependence was assessed with the participant self-rated Severity of Dependence Scale (SDS). The SDS has demonstrated acceptable internal consistency and validity in measuring subjective psychological dependence across various drugs and cultural contexts among PWUD. Dependence on individual drugs was defined using validated cut-off scores: ≥3 for cannabis, cocaine, cough syrup, or ketamine; ≥4 for MDMA; and ≥5 for opioids or methamphetamine (26–31).

COVID-19 infection and vaccination statuses of the participants were ascertained through a review of electronic medical records. Before 23 September 2022, Hong Kong residents with COVID-19 symptoms or a positive self-test result were legally mandated to undergo confirmatory polymerase chain reaction (PCR)-based nucleic acid testing via combined nasal and throat swabs or deep throat saliva samples to detect severe acute respiratory syndrome coronavirus 2 (SARS-CoV-2). Confirmed cases were obliged to remain in quarantine facilities or were admitted to designated public hospitals for treatment under the coordination of the Hong Kong Special Administrative Region (HKSAR) Government. Diagnoses of COVID-19 were recorded using ICD-10 codes U07.1 and U07.2 in a centralized medical record database. COVID-19 vaccination was provided free of charge by the HKSAR Government in a phased rollout beginning on 26 February 2021 (32), with vaccination records maintained in the same centralized system. Between 2020 and 2023, HKSAR residents were regarded as fully vaccinated if they had received at least two doses of a WHO-endorsed COVID-19 vaccine (33).

### Statistical analysis

Descriptive statistics were presented as proportions for categorical variables, and means (M) and standard deviations (SD) for all continuous variables. Differences between time points in primary drug use were tested using Stuart–Maxwell tests. Ordinal and logistic mixed-effects models were used to examine changes in four primary drug-use behaviors (frequency of drug use, avoidance of using drugs with others, sharing of drug-use equipment with others, and solitariness of drug use) across the three study time points and to identify the associated factors. Explanatory variables included time, level of worry about contracting COVID-19, severity of SUD, vaccination status (receipt of ≥2 doses), and laboratory-confirmed prior COVID-19 infection. For the severity of SUD, time was the only fixed effect modeled. Random intercepts were specified for each participant; no random slopes were included.

Time was contrast-coded using two orthogonal contrasts: the first compared T2 with T1 (T1 = −1/3, T2 = 2/3, T3 = −1/3), and the second compared T3 with T1 (T1 = −1/3, T2 = −1/3, T3 = 2/3). Continuous variables (level of worry about contracting COVID-19 and severity of SUD) were mean-centered. Binary variables (vaccination status and prior COVID-19 infection) were contrast-coded as “No” = −0.5 and “Yes” = 0.5. Demographic characteristics were not included as covariates because of model convergence issues. Drug type and comorbid psychiatric diagnosis were entered separately as covariates in the prediction models. Drug type reported at T1 was entered as a time-invariant baseline covariate. Each drug type was coded as a binary variable (used vs. not used). Drug types reported by fewer than 10 participants and comorbid psychiatric diagnoses represented by fewer than 20 participants were excluded from the models. No missing data were imputed, as the mixed-effects models yield unbiased estimates under the assumption that data are missing at random. Statistical significance was set at α = 0.05. All statistical analyses were conducted using R version 4.4.1 with the packages ordinal (version 2023.12–4.1), lme4 (version 1.1–35.5), and lmerTest (version 3.1–3).

## Results

A total of 130 PWUD (83.9% of 150 approached) aged 19–65 years (M = 40.03, SD = 11.04) who fulfilled the inclusion and exclusion criteria participated between March 2021 and January 2024. Retention rates were 75.4% (98/130) and 64.6% (84/130) at T2 and T3, respectively. Participants were predominantly male (72.3%) and ethnically Chinese (93.1%). Psychoactive substance-related psychotic disorder was the most prevalent psychiatric comorbidity (62.3%) (Table 1). Most participants reported single-drug use, with methamphetamine the most frequent primary drug across all time points (Table 2). Smoking was the predominant route of drug administration among participants. Overall, 88.5% of the participating PWUD had received ≥2 doses of a COVID-19 vaccine, and 27.7% had laboratory-confirmed COVID-19 infection (Supplementary Table 1).

**Table 1:** Demographics and the comorbid psychiatric disorders for the 130 participants at baseline (T1).

Baseline demographics and disorders	Details	*N* (%)
Gender	Male	94 (72.3)
	Female	36 (27.7)
Ethnicity	Chinese	121 (93.1)
	Non-Chinese	9 (6.9)
ICD-10 diagnosis for the comorbid psychiatric disorder	F00–F09 Organic, including symptomatic, mental disorders	2 (1.5)
	F1x.5 Mental and behavioral disorders due to psychoactive substance use, psychotic disorder	81 (62.3)
	F20–F29 Schizophrenia, schizotypal and delusional disorders	17 (13.1)
	F30–F39 Mood [affective] disorders	34 (26.2)
	F40–F48 Neurotic, stress-related and somatoform disorders	27 (20.8)
	F50–F59 Behavioral syndromes associated with physiological disturbances and physical factors	1 (0.8)
	F60–F69 Disorders of adult personality and behavior	7 (5.4)
	F80–F89 Disorders of psychological development	2 (1.5)
	F90–F99 Behavioral and emotional disorders with onset usually occurring in childhood and adolescence	8 (6.2)

*N*, frequency count; ICD-10, international classification of diseases, 10th Edition.

**Table 2:** Drug-use characteristics of participants across the three study time points.

Drug-use characteristic	T1 (*n* = 130) *N* (%)	T2 (*n* = 98) *N* (%)	T3 (*n* = 84) *N* (%)
Drug use in the previous 3 months#			
Methamphetamine	79 (60.8)	53 (54.1)	44 (52.4)
Cannabis	30 (23.1)	16 (16.3)	11 (13.1)
Cocaine	27 (20.8)	15 (15.3)	17 (20.2)
Cough syrup	26 (20.0)	18 (18.4)	12 (14.3)
Heroin	13 (10.0)	8 (8.2)	7 (8.3)
Ketamine	11 (8.5)	7 (7.1)	4 (4.8)
MDMA/ecstasy	1 (0.8)	1 (1.0)	0 (0.0)
GHB	4 (3.1)	4 (4.1)	0 (0.0)
LSD	1 (0.8)	0 (0.0)	0 (0.0)
Number of commonly used drugs			
1	82 (63.1)	72 (73.5)	57 (67.9)
2	35 (26.9)	19 (19.4)	16 (19.0)
≥3	13 (10.0)	4 (4.1)	2 (2.4)
Primary drug use*			
Methamphetamine	68 (52.3)	51 (52.0)	39 (46.4)
Cannabis	19 (14.6)	11 (11.2)	11 (13.1)
Cocaine	10 (7.7)	8 (8.2)	7 (8.3)
Cough syrup	22 (16.9)	16 (16.3)	14 (16.7)
Heroin	6 (4.6)	6 (6.1)	9 (10.7)
Ketamine	5 (3.8)	6 (6.1)	4 (4.8)
Primary method of drug administration			
Smoking	95 (73.1)	67 (68.4)	57 (67.9)
Oral	24 (18.5)	15 (15.3)	13 (15.5)
Nasal	6 (4.6)	4 (4.1)	3 (3.6)
Intravenous injection	5 (3.8)	0 (0.0)	0 (0.0)

*N*, frequency count; *n* = number of participants; T1, baseline [during the active COVID-19 pandemic period]; T2, after receipt of ≥2 doses of a WHO-endorsed COVID-19 vaccine; T3, after the WHO’s declaration of the end of the global COVID-19 pandemic. #: Participants could report more than one drug used during the preceding three months. *Primary drug was defined as the drug used most frequently during the preceding three months. MDMA, 3,4-methylenedioxymethamphetamine; GHB, gamma-hydroxybutyric acid; LSD, lysergic acid diethylamide.

At baseline (T1), 73.1% of participants met DSM-5 criteria for moderate-to-severe SUD and 70.0% had SDS-defined drug dependence. The majority (72.3%) reported drug use at least once per week (Table 3). Most PWUD (85.4%) reported no or only slight worry about contracting COVID-19. Smoking paraphernalia was the most common type of drug-use equipment shared (86.3%).

**Table 3:** Primary drug SUD, dependence and drug-use behaviors, and participants’ worry about contracting COVID-19 during the whole study period.

Outcomes	T1 (*n* = 130)	T2 (*n* = 98)	T3 (*n* = 84)
SUD severity			
No SUD	11 (8.5)	28 (28.6)	22 (26.2)
Mild SUD	24 (18.5)	23 (23.5)	22 (26.2)
Moderate SUD	48 (36.9)	19 (19.4)	17 (20.2)
Severe SUD	47 (36.2)	28 (28.6)	23 (27.4)
SDS			
Dependence	91 (70.0)	51 (52.0)	44 (52.4)
Frequency of use			
Abstinence for ≥3 months	0 (0.0)	23 (23.5)	24 (28.6)
Infrequent	4 (3.1)	7 (7.1)	3 (3.6)
Once a month	32 (24.6)	19 (19.4)	23 (27.4)
Once a week	17 (13.1)	9 (9.2)	9 (10.7)
Twice a week	34 (26.2)	18 (18.4)	12 (14.3)
Daily	43 (33.1)	22 (22.4)	13 (15.5)
Avoidance of using drugs with others	46 (35.4)	16 (21.3)	13 (21.6)
Sharing of drug-use equipment	73 (56.2)	38 (50.7)	23 (38.3)
Smoking tools	63 (86.3)	33 (86.8)	20 (87.0)
Drinking bottles	5 (6.8)	4 (10.5)	2 (8.7)
Snorting tools	4 (5.5)	1 (2.6)	1 (4.3)
Needles	1 (1.4)	0 (0.0)	0 (0.0)
Solitariness of drug use			
With others	38 (29.2)	27 (36.0)	24 (40.0)
Mainly with others	23 (17.1)	13 (17.3)	9 (15.0)
50:50	16 (12.3)	6 (8.0)	5 (8.3)
Mainly alone	21 (16.2)	7 (9.3)	7 (11.7)
Alone	32 (24.6)	22 (29.3)	15 (25.0)
Worry about contracting COVID-19			
Not at all	73 (56.2)	62 (63.3)	62 (73.8)
Slightly worried	38 (29.2)	24 (24.5)	17 (20.2)
Worried	13 (10.0)	10 (10.2)	5 (6.0)
Quite worried	6 (4.6)	2 (2.0)	0 (0.0)

n, number of participants; COVID-19 = coronavirus disease 2019; SUD, substance use disorder; SDS, Severity of Dependence Scale; T1, baseline [during the active COVID-19 pandemic period]; T2, after receipt of ≥2 doses of a WHO-endorsed COVID-19 vaccine; T3, after the WHO’s declaration of the end of the global COVID-19 pandemic.

SUD severity was significantly lower at T2 (odds ratio [OR] = 0.42, 95% CI [0.25, 0.69], *p* = 0.001) and T3 (OR = 0.39, 95% CI [0.23, 0.67], *p* = 0.001) compared with T1 (Table 4; Figure 1A). Frequency of drug use also declined significantly at both subsequent time points [T2: OR = 0.34, 95% CI (0.20, 0.57), *p* < 0.001; T3: OR = 0.23, 95% CI (0.13, 0.40), *p* < 0.001]. Sharing of drug-use equipment decreased significantly only at T3 [OR = 0.24, 95% CI (0.09, 0.60), *p* = 0.003]. No significant changes were observed in avoidance of drug use with others or solitariness of drug use (both *p* > 0.05) (Figures 1B–E). Greater worry about contracting COVID-19 was associated with significantly higher odds of avoiding drug use with others [OR = 2.44, 95% CI (1.57, 3.78), *p* < 0.001] and lower odds of drug-use equipment sharing [OR = 0.55, 95% CI (0.32, 0.96), *p* = 0.036]. Prior COVID-19 infection was associated with reduced frequency of drug use [OR = 0.53, 95% CI (0.29, 0.97), *p* = 0.038], whereas vaccination status showed no association (*p* > 0.05). Comorbid psychiatric diagnoses were not associated with any of the assessed drug-use behaviors (*p* > 0.05), and findings remained similar after adjustment for comorbid psychiatric diagnoses (Supplementary Table 2).

**Table 4:** Results of ordinal and logistic mixed-effects models for factors associated with changes in primary drug-use behaviors.

Factors	Severity of SUD			Frequency of drug use			Avoidance of using drugs with others			Sharing of drug-use equipment			Solitariness of drug use		
	OR	95% CI	*p*-value	OR	95% CI	*p*-value	OR	95% CI	*p*-value	OR	95% CI	*p*-value	OR	95% CI	*p*-value
T2 – T1	0.42	0.25–0.69	**0.001**	0.34	0.20–0.57	**<0.001**	0.49	0.23–1.05	0.065	0.62	0.28–1.36	0.232	0.88	0.48–1.61	0.671
T3 – T1	0.39	0.23–0.67	**0.001**	0.23	0.13–0.40	**<0.001**	0.55	0.24–1.25	0.154	0.24	0.09–0.60	**0.003**	0.59	0.30–1.18	0.135
Worry about contracting COVID-19	-	-	-	1.23	0.88–1.72	0.229	2.44	1.57–3.78	**<0.001**	0.55	0.32–0.96	**0.036**	1.00	0.65–1.53	0.995
Vaccination (receipt ≥2 doses of vaccine)	-	-	-	1.41	0.52–3.79	0.500	0.70	0.22–2.22	0.545	3.72	0.67–20.52	0.131	1.98	0.52–7.61	0.320
Prior COVID-19 infection	-	-	-	0.53	0.29–0.97	**0.038**	0.88	0.43–1.84	0.741	1.76	0.63–4.93	0.285	1.69	0.72–3.97	0.232

Bold values represent statistically significant results. OR, odds ratio; CI, confidence interval; SUD, substance use disorder; T1, baseline [during the active COVID-19 pandemic period]; T2, after receipt of ≥2 doses of a WHO-endorsed COVID-19 vaccine; T3, after the WHO’s declaration of the end of the global COVID-19 pandemic.

**Figure 1:**
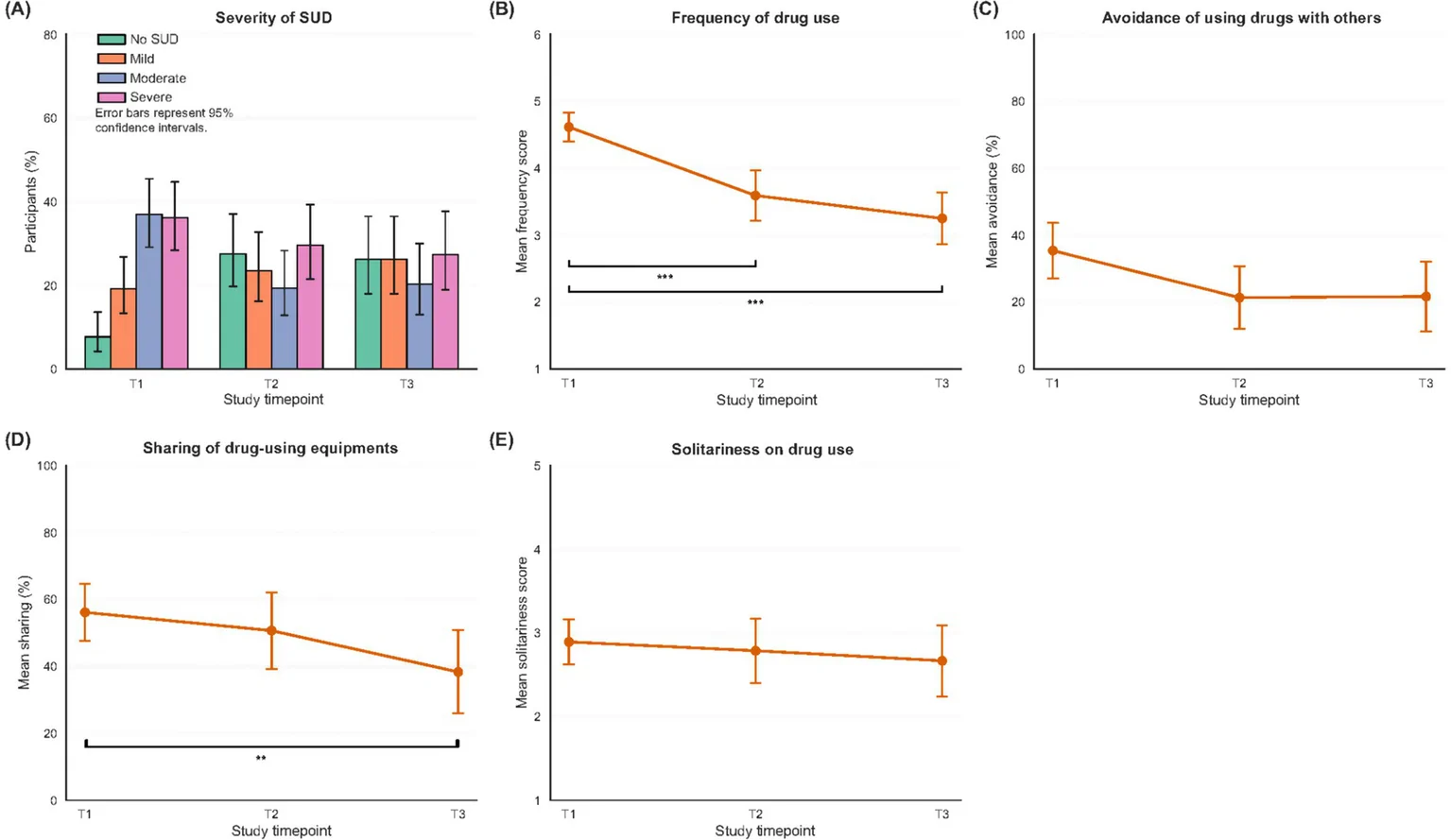
Severity of SUD and changes in primary drug-use behaviors across the three study time points. **(A–E)** represent the severity of SUD and changes in primary drug-use behaviors throughout the study period. **Indicates *p* < 0.01 and ***Indicates *p* < 0.001. SUD, substance use disorder; SDS, Severity of Dependence Scale; T1, baseline [during the active COVID-19 pandemic period]; T2, after receipt of ≥2 doses of a WHO-endorsed COVID-19 vaccine; T3, after the WHO’s declaration of the end of the global COVID-19 pandemic.

Primary drug use remained stable among participants over the three time points (all *p* > 0.05; Supplementary Table 3). Cough syrup was the only drug significantly associated with the examined drug-use behaviors. It was linked to a higher frequency of use [OR = 4.93, 95% CI (2.31, 10.53), *p* < 0.001] but lower likelihood of both solitary use [OR = 0.28, 95% CI (0.09, 0.82), *p* = 0.021] and sharing of drug-use equipment [OR = 0.21, 95% CI (0.05, 0.81), *p* = 0.024]. No significant associations were observed for other drugs (all *p* > 0.05), and results for drug-use behaviors remained consistent after adjustment for primary drug use (Supplementary Table 4).

## Discussion

This is the first prospective longitudinal study to examine changes in drug-use behaviors among PWUD with comorbid psychiatric disorders across the entire COVID-19 pandemic. Overall, participants showed reductions in drug-use frequency and SUD severity both during the pandemic and after the WHO declared the end of the pandemic, and they were less likely to share drug-use equipment after the pandemic. Greater worry about contracting COVID-19 and prior COVID-19 infection were both associated with safer drug-use behaviors. Cough syrup was the only drug significantly associated with more frequent use, less solitary use, and reduced sharing of drug-use equipment across the three study time points. No significant differences in drug-use behaviors were observed across comorbid psychiatric diagnosis. The laboratory-confirmed COVID-19 infection rate in this cohort (27.7%) was lower than the general population rate of 38.7% in Hong Kong during the same period (34, 35), consistent with previous findings that PWUD with psychiatric disorders were not at elevated risk of SARS-CoV-2 infection (18, 19).

Our finding of reduced drug-use frequency among PWUD with comorbid psychiatric disorders during the pandemic aligns with previous research linking pandemic-related restrictions to decreased drug use. From the onset of the COVID-19 pandemic until 28 December 2022, Hong Kong implemented stringent public health measures, including social isolation and border closures (36, 37). Restrictions on social gatherings and the closure of entertainment venues likely reduced opportunities for recreational and social drug use, contributing to the observed decline in frequency of drug use (36, 38–40). Border closures that disrupted drug supply chains may also have decreased drug availability and increased prices, thereby further lowering use (41–43). However, these effects were not uniform across countries. A national qualitative survey among PWUD in Canada reported an opposite pattern, with an increase in drug-use frequency attributed to border closures, unstable supply, and changes in drug potency and price (44). This contrast suggests that pandemic-related changes in drug use may also depend on the local drug market and social environment.

Importantly, participants’ drug-use frequency did not return to active-pandemic levels after the WHO declared the end of the pandemic in May 2023. Prior COVID-19 infection, which was found to be associated with reduced frequency of drug use in this study, may partly explain this sustained reduction. Unlike many other regions, confirmed infections and related mortality in Hong Kong rose exponentially only between 2022 and 2023 during the SARS-CoV-2 Omicron BA.2 wave (43). This delayed local epidemic may have contributed to the persistence of lower drug-use frequency even after the relaxation of pandemic public health measures.

Greater worry about contracting COVID-19 was associated with safer drug-use behaviors, including greater avoidance of using drugs with others and reduced sharing of drug-use equipment. However, it was not associated with frequency or solitariness of drug use. These associations may reflect a greater sense of agency and autonomy, whereby participants sought to protect their health by reducing the risk of infection (45). The absence of significant associations with frequency and solitariness is consistent with previous findings that worry exerts only a small effect on drug-use frequency, which may be moderated by the COVID Disregard Syndrome (46). Overall, the observed safer drug-use behaviors appear to reflect efforts to minimize infection-related risk rather than a comprehensive reduction in all forms of drug-related harm, such as the elevated risk of fatal overdose resulting from solitary use (47).

Among all the drugs examined, cough syrup was the only drug associated with more frequent use, yet less solitary use and reduced sharing of drug-use equipment. Cough syrup misused in Hong Kong commonly contains codeine (48). These findings are consistent with reports of increased prescription opioid use and the polarization of drug use toward more readily and legally available drugs during the pandemic (49, 50). Furthermore, because SARS-CoV-2 is transmitted primarily through respiratory droplets, participants may have been less likely to share bottles during the pandemic.

This study has several limitations. First, it focused on a specific clinical population of PWUD with comorbid psychiatric disorders. This limits generalizability to community-dwelling PWUD without psychiatric disorders and may not have fully captured changes in drug-use behaviors in that population. Second, the small sample size and high attrition by the final assessment may have reduced statistical power and incurred type II errors. Third, although PWUD in this study were asked to report their drug-use behaviors over the preceding three months at each time point, the nearly three-year study period may have introduced potential recall bias. Finally, because all participants were recruited from substance-use treatment clinics, any affective symptoms or worsening of drug-use behaviors that emerged during the pandemic may have been identified and managed before study enrollment. Clinicians and professionals working with PWUD should therefore interpret the findings with caution, particularly when applying the results of this study to formulate drug rehabilitation and care planning for PWUD with comorbid psychiatric disorders.

## Conclusion

In conclusion, this longitudinal cohort study provides novel insight into how PWUD with comorbid psychiatric disorders modified their drug-use behaviors throughout and beyond the COVID-19 pandemic. Our findings suggest that pandemic-related behavioral adaptations among this high-risk population were driven primarily by infection-related concerns and local public health restrictions rather than by vaccination status or specific comorbid psychiatric diagnoses. Clinicians and healthcare policymakers should remain vigilant regarding the potential for prescription drug misuse, particularly of codeine-containing cough syrup, both during and after public health emergencies. Sustained efforts to reinforce the observed reductions in drug-use frequency and equipment sharing may help support safer drug-use practices among PWUD with psychiatric comorbidity in the post-pandemic era (51).

## Data Availability

Individual participant data that underlie the results reported in this article after de-identification and the study protocol will be made available beginning 9 months and ending 36 months following the article publication to investigators whose proposed use of the data has been approved by an independent review committee for individual participant data meta-analysis. Proposals should be directed to AKKC and data access would follow the requirements laid in the Policy on the Management of Research Data and Records by the University of Hong Kong.
