# Supplementary material for: Changes in drug-use behaviors among people who use drugs with comorbid psychiatric disorders across the COVID-19 pandemic: a three-phase longitudinal cohort study
Source: Front Public Health. 2026 Sep 17;14:1878232. doi: 10.3389/fpubh.2026.1878232 (PMC13626888; doi:10.3389/fpubh.2026.1878232)
Supplement: Supplementary file 1 [file Table_1.docx]

**Supplementary Materials: Changes in drug-use behaviors among people who use drugs with comorbid psychiatric disorders across the COVID-19 pandemic: a three-phase longitudinal cohort study**

Supplementary Table 1. COVID-19 vaccination and infection statuses for the 130 participants during the study period.

Supplementary Table 2. Results from regression analysis on primary drug-use behaviors adjusted for ICD-10 comorbid psychiatric disorders.

Supplementary Table 3. Pairwise changes in participants’ primary drug use across the three study time points.

Supplementary Table 4. Results from regression analysis on drug-use behaviors adjusted for primary drug use.

Supplementary Table 1. COVID-19 vaccination and infection statuses for the 130 participants during the study period.

|  |  | N (%) |
| --- | --- | --- |
| Vaccination status | No vaccination | 12 (9.2) |
|  | One dose | 3 (2.3) |
|  | Two doses | 14 (10.8) |
|  | Three doses | 86 (66.2) |
|  | ≥4 doses | 15 (11.5) |
| Laboratory-confirmed COVID-19 infection | Infected  Never infected | 36 (27.7)  94 (72.3) |
|  |  |  |

N = frequency count; COVID-19 = coronavirus disease 2019.

Supplementary Table 2. Results from regression analysis on primary drug-use behaviors adjusted for ICD-10 comorbid psychiatric disorders.

|  | Frequency of drug use | | | Avoidance | | | Sharing | | | Solitariness | | |
| --- | --- | --- | --- | --- | --- | --- | --- | --- | --- | --- | --- | --- |
|  | OR | 95%CI | P | OR | 95%CI | P | OR | 95%CI | P | OR | 95%CI | P |
| T2 – T1 | 0.34 | 0.20 – 0.57 | **<0.001** | 0.49 | 0.23 – 1.04 | 0.062 | 0.63 | 0.29 – 1.38 | 0.247 | 0.87 | 0.47 – 1.60 | 0.653 |
| T3 – T1 | 0.23 | 0.13 – 0.40 | **<0.001** | 0.55 | 0.24 – 1.26 | 0.161 | 0.24 | 0.09 – 0.62 | **0.003** | 0.59 | 0.30 – 1.16 | 0.126 |
| Worry about contracting COVID-19 | 1.23 | 0.88 – 1.72 | 0.229 | 2.36 | 1.52 – 3.65 | **<0.001** | 0.56 | 0.32 – 0.98 | **0.041** | 0.99 | 0.64 – 1.51 | 0.951 |
| Vaccination (receipt ≥2 doses of vaccine) | 1.41 | 0.52 – 3.79 | 0.500 | 0.69 | 0.22 – 2.19 | 0.531 | 3.60 | 0.65 – 19.84 | 0.142 | 2.09 | 0.54 – 8.04 | 0.282 |
| Prior COVID-19 Infection | 0.53 | 0.29 – 0.97 | **0.038** | 0.88 | 0.42 – 1.83 | 0.726 | 1.70 | 0.60 – 4.81 | 0.315 | 1.77 | 0.75 – 4.17 | 0.192 |
| Mental and behavioral disorders due to psychoactive substance use, psychotic disorder | 0.84 | 0.48 – 1.46 | 0.535 | 1.14 | 0.57 – 2.28 | 0.711 | 0.69 | 0.26 – 1.82 | 0.450 | 1.23 | 0.55 – 2.76 | 0.607 |
| Mood [affective] disorders | 0.58 | 0.31 – 1.06 | 0.076 | 0.95 | 0.43 – 2.08 | 0.891 | 1.22 | 0.41 – 3.66 | 0.717 | 0.73 | 0.29 – 1.82 | 0.497 |
| Neurotic, stress-related and somatoform disorders | 1.63 | 0.83 – 3.20 | 0.155 | 1.43 | 0.64 – 3.17 | 0.380 | 1.17 | 0.37 – 3.70 | 0.789 | 0.82 | 0.31 – 2.12 | 0.678 |

Note: bold values represent statistically significant results. OR = odds ratio, CI = confidence interval, p = p-value; SUD = substance use disorder; T1 = baseline [during the active COVID-19 pandemic period]; T2 = after receipt of ≥2 doses of a WHO-endorsed COVID-19 vaccine; T3 = after the WHO’s declaration of the end of the global COVID-19 pandemic.

| Supplementary Table 3. Pairwise changes in participants’ primary drug use across the three study time points. | | | | | | | | |  |
| --- | --- | --- | --- | --- | --- | --- | --- | --- | --- |
| **Comparison** | **Paired n** | **Chi-square** | **Unchanged n** | **Changed n** | **Unchanged %** | **df** | **Raw p-value** | **Holm-adjusted p-value** |  |
| T1 vs T2 | 98 | 6.818 | 85 | 13 | 86.7 | 5 | 0.235 | 0.688 |  |
| T2 vs T3 | 70 | 6.638 | 57 | 13 | 81.4 | 5 | 0.249 | 0.688 |  |
| T1 vs T3 | 84 | 6.886 | 66 | 18 | 78.6 | 5 | 0.229 | 0.688 |  |
| Pairwise comparisons were conducted using Stuart–Maxwell tests of marginal homogeneity. Percentages of unchanged primary drug were calculated among participants with primary drug data at both time points in each comparison. Holm adjustment was applied to account for the three pairwise comparisons. n: number of participants; df = degrees of freedom; T1 = baseline [during the active COVID-19 pandemic period]; T2 = after receipt of ≥2 doses of a WHO-endorsed COVID-19 vaccine; T3 = after the WHO’s declaration of the end of the global COVID-19 pandemic. | | | | | | | | |  |
|  |  |  |  |  |  |  |  |  |  |
|  |  |  |  |  |  |  |  |  |  |
|  |  |  |  |  |  |  |  |  |  |

Supplementary Table 4. Results from regression analysis on drug-use behaviors adjusted for primary drug use.

|  | Frequency of drug use | | | Avoidance | | | Sharing | | | Solitariness | | |
| --- | --- | --- | --- | --- | --- | --- | --- | --- | --- | --- | --- | --- |
|  | OR | 95%CI | P | OR | 95%CI | P | OR | 95%CI | P | OR | 95%CI | P |
| T2 – T1 | 0.34 | 0.20 – 0.57 | **<0.001** | 0.49 | 0.23 – 1.05 | 0.066 | 0.64 | 0.29 – 1.43 | 0.277 | 0.91 | 0.50 – 1.68 | 0.767 |
| T3 – T1 | 0.23 | 0.13 – 0.40 | **<0.001** | 0.54 | 0.23 – 1.23 | 0.143 | 0.22 | 0.08 – 0.57 | **0.002** | 0.60 | 0.30 – 1.19 | 0.142 |
| Worry about contracting COVID-19 | 1.23 | 0.88 – 1.72 | 0.229 | 2.47 | 1.58 – 3.85 | **<0.001** | 0.57 | 0.33 – 0.99 | **0.045** | 1.05 | 0.69 – 1.61 | 0.809 |
| Vaccination (receipt ≥2 doses of vaccine) | 1.41 | 0.52 – 3.79 | 0.500 | 0.77 | 0.24 – 2.49 | 0.664 | 4.29 | 0.79 – 23.44 | 0.093 | 2.47 | 0.65 – 9.39 | 0.184 |
| Prior COVID-19 infection | 0.53 | 0.29 – 0.97 | **0.038** | 0.89 | 0.42 – 1.89 | 0.756 | 2.04 | 0.71 – 5.80 | 0.183 | 1.75 | 0.75 – 4.08 | 0.195 |
| Meth- amphetamine | 0.77 | 0.42 – 1.44 | 0.420 | 1.24 | 0.54 – 2.88 | 0.615 | 2.82 | 0.87 – 9.18 | 0.084 | 1.86 | 0.74 – 4.68 | 0.186 |
| Cannabis | 1.48 | 0.78 – 2.79 | 0.230 | 0.61 | 0.25 – 1.51 | 0.290 | 2.43 | 0.73 – 8.14 | 0.148 | 1.68 | 0.65 – 4.33 | 0.280 |
| Cocaine | 1.78 | 0.92 – 3.44 | 0.085 | 1.05 | 0.43 – 2.60 | 0.915 | 1.82 | 0.53 – 6.27 | 0.341 | 1.01 | 0.37 – 2.71 | 0.992 |
| Cough syrup | 4.93 | 2.31 – 10.53 | **<0.001** | 0.98 | 0.39 – 2.52 | 0.974 | 0.21 | 0.05 – 0.81 | **0.024** | 0.28 | 0.09 – 0.82 | **0.021** |
| Heroin | 1.58 | 0.69 – 3.65 | 0.282 | 1.15 | 0.39 – 3.40 | 0.795 | 0.62 | 0.14 – 2.65 | 0.516 | 1.73 | 0.52 – 5.83 | 0.374 |
| Ketamine | 1.00 | 0.40 – 2.50 | 0.998 | 0.93 | 0.25 – 3.42 | 0.914 | 1.75 | 0.30 – 10.37 | 0.536 | 1.04 | 0.25 – 4.29 | 0.956 |

Note: bold values represent statistically significant results. OR = odds ratio, CI = confidence interval, p = p-value; SUD = substance use disorder; T1 = baseline [during the active COVID-19 pandemic period]; T2 = after receipt of ≥2 doses of a WHO-endorsed COVID-19 vaccine; T3 = after the WHO’s declaration of the end of the global COVID-19 pandemic.
